# Maximal Time Spent at VO_2max_ from Sprint to the Marathon

**DOI:** 10.3390/ijerph17249250

**Published:** 2020-12-10

**Authors:** Claire A. Molinari, Johnathan Edwards, Véronique Billat

**Affiliations:** 1 Unité de Biologie Intégrative des Adaptations à l’Exercice, Université Paris-Saclay, Univ Evry, 91000 Evry-Courcouronnes, France; contact@billatraining.com; 2 BillaTraining SAS, 32 rue Paul Vaillant-Couturier, 94140 Alforville, France; 3 Faculté des Sciences de la Motricité, Unité d’enseignement en Physiologie et Biomécanique du Mouvement, 1070 Bruxelles, Belgium; jjedwards505@gmail.com

**Keywords:** VO_2max_, performance, running

## Abstract

Until recently, it was thought that maximal oxygen uptake (VO_2max_) was elicited only in middle-distance events and not the sprint or marathon distances. We tested the hypothesis that VO_2max_ can be elicited in both the sprint and marathon distances and that the fraction of time spent at VO_2max_ is not significantly different between distances. Methods: Seventy-eight well-trained males (mean [SD] age: 32 [13]; weight: 73 [9] kg; height: 1.80 [0.8] m) performed the University of Montreal Track Test using a portable respiratory gas sampling system to measure a baseline VO_2max_. Each participant ran one or two different distances (100 m, 200 m, 800 m, 1500 m, 3000 m, 10 km or marathon) in which they are specialists. Results: VO_2max_ was elicited and sustained in all distances tested. The time limit (Tlim) at VO_2max_ on a relative scale of the total time (Tlim at VO_2max_%Ttot) during the sprint, middle-distance, and 1500 m was not significantly different (*p* > 0.05). The relevant time spent at VO_2max_ was only a factor for performance in the 3000 m group, where the Tlim at VO_2max_%Ttot was the highest (51.4 [18.3], r = 0.86, *p* = 0.003). Conclusions: By focusing on the solicitation of VO_2max_, we demonstrated that the maintenance of VO_2max_ is possible in the sprint, middle, and marathon distances.

## 1. Introduction

Classically, the solicitation of the maximal uptake of oxygen (VO_2max_) was thought only to be possible in the middle-distance (1500 m) events, and not the sprint or the marathon distances [1]. (1) Power output may be high (greater than critical speed), but insufficient to elicit VO_2max_ (i.e., the average marathon speed). (2) Power may be very high or maximal, and sufficient to drive VO_2_ to its maximum before exhaustion (i.e., middle-distance events). (3) Power may be extremely high, such that the subject becomes exhausted before sufficient time has elapsed for VO_2_ to reach its maximum (i.e., sprint events) [2].

This classification is the basis of the century-old constant-speed paradigm applied in laboratories since the discovery of VO_2max_ by AV Hill in 1923 [3]. Today, innovative technologies such as the portable breath-by-breath gas exchange systems allows researchers to investigate the solicitation of VO_2max_ during 100 and 200 m sprints in elite runners. By assessing the fundamental physiology, it has been shown that the change in tissue oxygen uptake is directly proportional to changes in creatine (Cr) content [4]. This close reciprocal relationship between pulmonary VO_2_ and phosphocreatine (Pcr) has been demonstrated at the systemic level during high-intensity constant power output exercises [5]. Hence, there is a close relationship between oxygen uptake kinetics and changes in Cr/Pcr ratios. The rapid depletion of creatine phosphate during a sprint may be a signal for a rapid increase in VO_2_ and possibly until VO_2max_. Therefore, our first hypothesis is that VO_2max_ can be reached during a sprint, but also that the relative time spent at VO_2max_ may be of the same order during middle distances, and possibly a discriminant factor of performance.

The marathon is the longest Olympic endurance distance. Previous research has estimated that the marathon only elicits a fractional utilization of VO_2max_ [6]. However, technological advances now allow breath-by-breath VO_2_ measurements during an entire marathon. In the past, it was only possible to measure VO_2_ over 1 or 2 km using Douglas bags from the back of a moving vehicle, as performed by Michael Maron. These pioneering experiments highlighted marathon training and performance, as he showed that VO_2max_ was reached during the marathon and our research confirms his results. Indeed, the paradigm of constant (constant vs average) velocity still endures today as determined by the ratio of energy output and the cost of running [6]; this all comes from the treadmill experiments of constant speed physiology. It is generally thought that VO_2max_ is not elicited in the marathon and that it must be run below maximal aerobic speed (vVO_2max_) in order to maintain a sub lactate threshold VO_2_ steady state [7,8]. One obvious consequence of the slow component response is that it creates a range of velocities, all which elicit VO_2max_, provided the exercise is continued to exhaustion. VO_2max_ can be elicited during constant power exercise, over a range of intensities that may be higher or lower than the minimum value for which it occurs during incremental exercise [9]. Maron’s pioneering research reported that VO_2max_ could be elicited during a marathon; however, we did not have portable gas exchange measurements to confirm this remarkable result [10]. Today, portable breath-by-breath gas exchange analyzers have minimal measurement delays and can be easily worn in competition.

The plateau in VO_2_ at the end of an incremental exercise test is used as an important criterion to validate that VO_2max_ has been achieved [6]; however, the duration that subjects can sustain that plateau has largely been ignored. The time limit at PVO_2max_ (Tlim@PVO_2max_), while reproducible, has been reported to be highly variable between subjects (3–8 min) [11]; it is negatively correlated with PVO_2max_ and VO_2max_ but positively correlated with the maximal oxygen deficit, which is an index of the ability to generate energy from anaerobic metabolism (i.e., anaerobic capacity) [12,13]. Hence, while debates continue around the central versus peripheral limiting factors of VO_2max_ [14,15], the limiting factors of VO_2max_ and of the ability to sustain VO_2max_ remain to be investigated independently of PVO_2max_ [13]. It was shown that VO_2max_ can be sustained for a longer duration when exercise is controlled by the maintenance of VO_2max_, and that the limiting cardiovascular factors of endurance at VO_2max_ are unrelated to its value.

The examination of the time limit at VO_2max_ in different running events is a more ecological approach to the time to plateau at VO_2max_ as it relates to the total time run from sprint to the marathon. Real-world races are not run at constant speeds [16,17], and we wish to reverse the paradigm of power around PVO_2max_ or constant VO_2_ in order to examine the plateau at VO_2max_ as a common performance factor when expressed as a percentage of total race time. Indeed, the underlying idea is that the greater the energy at VO_2max_ (maximum oxidation rate), the more Adenosine Triphosphate resynthesized from creatine and lactic acid contributes to sprint and marathon performances. Hence, the more relative time run at VO_2max_, the better the performance, independent of the distance. The concept of relative time to exhaustion at VO_2max_ could be a central energy concept independent of whether the dominant metabolism is aerobic or anaerobic. We hypothesize that this concept could lead to a new method of high intensity interval training that uses very short sprints around the average marathon speed in accordance with the target distance (from 100 to 42,195 m).

Therefore, our primary hypothesis is that VO_2max_ can be sustained from the sprint to the marathon and independent of the distance run, the time spent relative to exhaustion at VO_2max_, as expressed as a percentage of the total performance time, is a discriminant factor for performance.

## 2. Materials and Methods

Seventy-eight well-trained male athletes (training 4 days per week) participated in the study (mean ± standard deviation [SD] age: 32 [13]; weight: 73 [9] kg; height: 1.80 [0.8 m]. The participants’ preferred racing distances were as follows: 100 m (*n* = 13), 200 m (*n* = 13), 800 m (*n* = 8), 1500 m (*n* = 16), 3000 m (*n* = 9), 10 km (*n* = 7), and the marathon (*n* = 12). All of the participants were experienced in their respective full effort race distances and VO_2max_ tests (University of Montreal Track Test, UMTT). All subjects gave their informed consent for inclusion before they participated in the study. The study was conducted in accordance with the Declaration of Helsinki, and the protocol was approved by an independent ethics committee (CPP Sud-Est V, Grenoble, France; reference: 2018-A01496-49). All participants were provided with study information and gave their written consent before participation.

All participants performed the University of Montreal Track Test (UMTT), to determine individual VO_2max_ values. After 7 to 14 days, they ran one or two different race simulation efforts in which they are specialists (100 m, 200 m, 800 m, 1500 m, 3000 m, 10 km or the marathon). A portable breath-by-breath sampling system (K5 [18], COSMED Srl, Rome, Italy) that continuously measured respiratory gases (oxygen uptake [VO_2_], ventilation [VE], and the respiratory exchange ratio) was worn in both the UMTT and race efforts. During the 7 to 14 day period between the UMTT and the running effort, the participants were instructed to continue their training activities as normal. A global positioning system watch (Garmin, Olathe, KS, USA) was used to measure the heart rate and the speed responses (5 s averaged data) of each effort. In the UMTT, the rating of perceived exertion (RPE), on a scale from 6 (least exertion) to 20 (greatest exertion) [19], was recorded 15 s before the end of each stage [20].

### 2.1. Determination of Maximal Oxygen Uptake and Velocity Associated with VO_2_max—The UMTT

The UMTT was conducted on a 400 m track with cones placed every 20 m. Pre-recorded sound beeps indicated when the subject needed to be near a cone to maintain the imposed speed. A longer sound marked speed increments. The first step was set to 8.5 km·h^−1^, with a subsequent increase of 0.5 km·h^−1^ every minute. When the runner was unable to maintain the imposed pace and thus failed to reach the cone in time for the beep on two consecutive occasions, the test was terminated. The speed corresponding to the last completed step was recorded as the vVO_2max_ (km·h^−1^). During the UMTT, VO_2max_ was confirmed by a visible plateau in VO_2_ (≤2 mL·kg^−1^·min^−1^) with a standard increase in exercise intensity, and any indicative secondary criteria (visible signs of exhaustion; HRmax ±10 beats·min^−1^) around the point of volitional exhaustion and an RPE of 19–20.

### 2.2. Determination of The Time Limit at VO_2max_ (Tlim at VO_2max_)

Oxygen uptake is not a simple function of power output or velocity, for it is a function of time as well. Even steady-state oxygen uptake is not a linear function of power output beyond a certain level [2]. The slow component of oxygen uptake and increasing oxygen cost of exercise at higher powers outputs complicates the issue [21]. The slow component has, however, been successfully modeled, both theoretically [22] and empirically [23], and the energy cost of running can safely be assumed to be constant (or very nearly so) provided the power or velocity range is narrow [2]. Perhaps, then, these difficulties can be largely overcome by considering endurance at a fixed value of oxygen uptake, say at its maximum (VO_2max)_ [2]. This time limit at VO_2max_ depends on the duration of the subject’s exhaustion time (time limit = Tlim) and the time to reach VO_2max_ (TA VO_2max_), both of which decrease with increasing exercise intensity (Tlim VO_2max_ = Tlim − TA VO_2max_) [12]. Steady-state VO_2_ was defined when the subject reached 95% of incremental VO_2max_ [12] during an incremental test. During each race effort, the VO_2max_ Tlim was therefore computed by calculating the difference between the total running time (Tlim) and the time taken to reach 95% incremental VO_2max_ (TA VO_2max_) [12].

Tlim at VO_2max_ is also defined as the time (seconds) spent at maximal oxygen consumption during the completed distance. Knowing that VO_2max_ was the maximal oxygen consumption during the UMTT (mL·kg^−1^·min^−1^), we then processed the data to test the effect of the Tlim VO_2max_ on the relative exercise duration for each distance. We normalized the duration of the run on a relative scale of total time (%Ttot) by comparing the time to the distance. For each effort, the Tlim at VO_2max_, (assuming that VO_2max_ was reached and maintained) is the Tlim at VO_2max_%Ttot and is determined to be the ratio between Tlim at VO_2max_ and total time of the effort.

### 2.3. Calculation of the Intensity of Race in the Percentage of Vvo_2max_ (Intensity of Exercise %Vvo_2max_)

We also calculated exercise intensity (average speed) as a percentage of vVO_2max_ (km·h^−1^), since it would appear that the factors limiting time spent at VO_2max_ are different depending on whether the intensity is greater or less than vVO_2max_ [13].

### 2.4. Statistical Analysis

All statistical analyses were performed using XLSTAT software (version 1 January 2019, Addinsoft, Paris, France). For each variable, the normality and homogeneity of the data distribution were examined using a Shapiro–Wilk test. A one-way analysis of variance (ANOVA) was applied to assess the various race distances in terms of performance variables: International Association of Athletics Federations (IAAF) score, running time (s), vVO_2max_ (km·h^−1^), VO_2max_ (mL·kg^−1^·min^−1^), and post-run blood lactate level (mM). A one-way analysis of variance (ANOVA) was also used to assess the time at VO_2max_ and the intensity of exercise. Pearson’s coefficient (r) was used to measure the correlations between performances, Tlim at VO_2max_%Ttot, and intensity of exercise %vVO_2max_.

## 3. Results

The descriptive physiological responses in UMTT are summarized in Table 1. Sprinters and 800 m runners have significantly lower VO_2max_ than the middle- and long-distance runners (3000 m and 10 km) (Table 1). There were significant differences in VO_2max_ between participants who ran the 800 m and those who ran the sprints, 3000 m, and 10 km (*p* < 0.0001, *p* < 0.0001, and *p* = 0.0002, respectively). VO_2max_ was significantly higher in the participants who ran the 10 km than in the sprinters and the 3000 m runners (*p* < 0.0001 and *p* < 0.0001, respectively).

**Figure 1:**
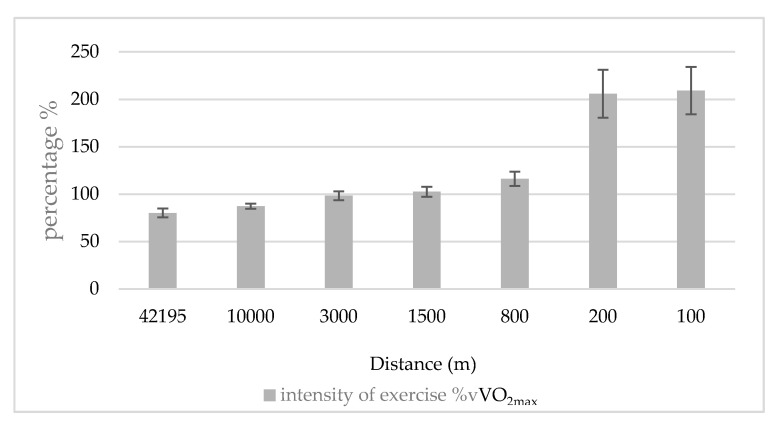
Exercise intensity (average speed) as a percentage of vVO_2max_ at each race.

The 100, 200, and 800 m were run at much higher values than their vVO_2max_ (209 ± 25, 206 ± 25, and 116 ± 8% of vVO_2max_, respectively. *p* < 0.001). All other distances were run at or below vVO_2max_, 102, and 80% of vVO_2max_ in the 1500 m and the marathon, respectively (Figure 1).

**Figure 2:**
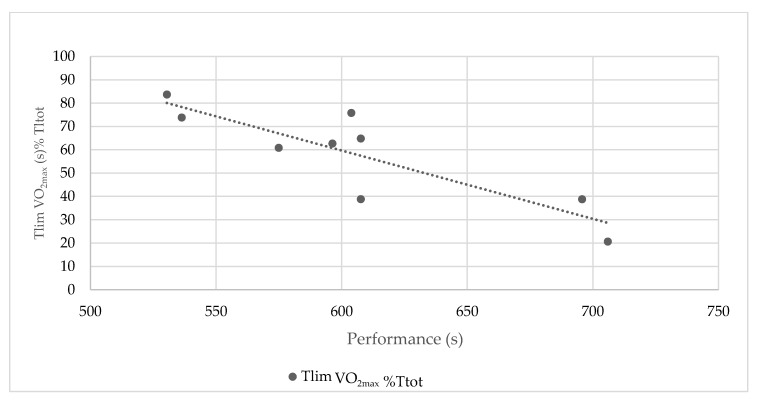
Correlation between the Tlim VO_2max_ on the relative exercise duration (Tlim at VO_2max_%Ttot) and the performance in the 3000 m race effort.

Due to the large difference in relative speed to vVO_2max_, Tlims at VO_2max_%Ttot during the sprint, middle-distance, 800 m, and the 1500 m were not significantly different (Table 2). The highest Tlim at VO_2max_%Ttot was measured in the 3000 m race, while the lowest was measured in the marathon (Figure 2). The 3000 m runners spent their half of the time at VO_2max_ (51 ± 18% of Ttot), while all of the marathon runners all reached VO_2max_, but only for 5% of the time (Table 2).

The relative time spent at VO_2max_ was only a factor predicting performance in the groups for which the Tlim at VO_2max_%Ttot was the highest and the lowest, the 3000 m and the marathon, respectively. Indeed, the 3000 m race was the distance eliciting the highest Tlim at VO_2max_%Ttot (more than half of the effort) and the distance for which the Tlim at VO_2max_%Ttot was significantly correlated with the performance (r = 0.86, *p =* 0.003, Figure 2).

Seventy-four percent of the 3000 m performance variance could be predicted by the relative time limit at VO_2max_ (Tlim at VO_2max_%Ttot), higher than with vVO_2max_ (69%). Furthermore, as highlighted above, even if the relative time spent at VO_2max_ was low (5%) during the marathon, the fraction of vVO_2max_ was a significant predictor of marathon performance (r² = 0.81).

## 4. Discussion

Classically, it was thought that neither the sprint nor the marathon elicited VO_2max_. Our results show that VO_2max_ can be elicited and sustained in the sprint, marathon, and middle-distance events. Furthermore, we found that the time spent at VO_2max_ represents a high fraction of the distance run in the sprint and middle-distances (800–3000 m). However, this time spent at VO_2max_ was only correlated with the 3000 m event.

We believe that this is the first study focusing on the solicitation of VO_2max_ during the sprint (100, 200 m). The solicitation of VO_2max_ is brief, given that both oxygen kinetics and the delay of achieving VO_2max_ depends heavily on the acceleration phase [24]. Indeed, the time constant values of the fundamental amplitude for VO2, the muscle phosphocreatine response to exercise, and VO_2_ dynamics cohere during both the moderate and high-intensity exercise [25].

We showed that VO_2max_ is elicited in the marathon, even though the time spent at VO_2max_ is only 5 percent. The results reported by Michael Maron (1976) agree with our results. Even if the Tlim at VO_2max_%Ttot was the lower in the marathon (4 ± 4%), most marathon runners reached VO_2max_ during the effort in Maron’s study.

The relative time runners spent at VO_2max_ were not significantly different between the sprint and short middle-distance events (800 and 1500 m).

Our group of elite national level sprinters possess an exceptionally high maximal aerobic capacity that must be considered when examining our results [26]. Indeed, this ability to rapidly reach VO_2max_ during a sprint allows an athlete to perform sprint repeats during training and racing [27]. In a recent study, the authors investigated the aerobic contribution to isolated sprints within a repeated-sprint bout involving 5 × 6 s sprints [28]. The findings have shown that the aerobic contribution to the first sprint is ∼10%, while during the fifth sprint, it is ∼40%. The aerobic contribution to the final sprint of each bout was also significantly related to VO_2max_ [28]. This is supported by the VO_2_ attained during the final sprint of each bout, which was not different from VO_2max_ (*p* = 0.448). Due to the incomplete recovery between sprints, it is possible that the progressive increases in PCr breakdown and Pi accumulation over the course of the 5 × 6 s sprints would also have driven the increase in VO_2_ from the first to the final sprint [28]. Thus, the significantly greater VO_2_ in the fifth sprint of each bout can probably be attributed to starting from an elevated baseline [29], priming as a consequence of the previous sprints, and an ADP-mediated stimulation of VO_2_ [28]. Their findings suggest that the aerobic contribution to repeated-sprint exercise may be limited by VO_2max_ and that by increasing this capacity a greater aerobic contribution may be achieved during latter sprints, potentially improving performance [28,29]. it is likely that all sprints after the first were initiated from an elevated baseline [30], which would have elevated the VO_2_ during subsequent sprints [28]. Aerobic metabolism provides nearly 50% of the energy during the second sprint of 10 or 30 s, whereas the phosphocreatine (PCr) availability is essential for high power output during the initial 10 s [27]. Peak oxygen deficit is also an important factor of performance in the sprint and middle-distance events. Furthermore, multiple regression analyses indicate that the peak oxygen deficit is the strongest metabolic predictor of performance in the 800, 1500, and 5000 m events [31].

Likewise, Billat et al. reported that a high peak oxygen consumption and the ability to run fast over a 1000 m section of the marathon determined the difference between an elite marathon performance (2 h 6 min–2 h 11 min) and a non-elite marathon time (2 h 12 min–2 h 16 min) [32].

Force-velocity characteristics and maximal anaerobic power are of great interest, especially in elite runners [33].

Successful elite runners possess the ability to run at high speeds over periods of a few seconds to several minutes [34]. This is likely mediated by the ability to rapidly deplete phosphocreatine (PCr) [28], accelerate the oxygen kinetics, and increase the relative time spent at VO_2max_. Indeed, evidence suggests that PCr depletion is related to sprint duration and subjects’ training status [35]. Hirvonen et al. (1987) suggested that sprint performance is related to depleting a more significant amount of high-energy phosphates and at faster rates during the initial stages of exercise; he demonstrated that PCr depletion was greater in a group of elite national level 100 m track sprinters [36]. The elite sprinters depleted significantly higher amounts of PCr than the slower sprinters during 80 and 100 m sprints (76 and 71%) [36]. The rapid depletion of PCr could also induce faster oxygen kinetics and, therefore, a more extended time spent at VO_2max_. Korzeniewski and Zoladz (2004) (this last one being a prior high 800 m level) clearly demonstrated that the half–transition time of VO_2_ kinetics is determined by the amount of PCr that has been transformed into creatine during the rest-to-work transition [37].

A fast-start during a running effort has been reported to increase VO_2_ kinetics and to improve exercise tolerance [38,39,40]. Sahlin (2004) highlighted that the ATP turnover rate during a 100 m sprint is estimated to be three-fold higher than during a marathon and 50 times higher than at rest [41]. Acceleration corresponds to about 10 and 40% of the total energy demand during 400 and 100 m running, respectively [41]. During a 5000 m effort, Sahlin (2004) considered that the total energy demand is significant, and that the contribution from kinetic energy becomes negligible. If we consider that the time to reach VO_2max_ contributes to the relative time spent at VO_2max_, our results show that until the 10 km, the time spent at VO_2max_ is not negligible (50% on 3000 m and 31% on 10 km).

Furthermore, once VO_2max_ is reached in a sprint to the 10 km, it is maintained until the end of the effort, and this contributes to the relative time to exhaustion at VO_2max_. This contrasts with prior studies that found a systematic decrease in VO_2_ in the last 100 m of a 400 and 800 m effort after VO_2max_ was reached, but they did not observe this systematic decrease at the end of the 1500 m effort [42]. We can explain this difference in VO_2_ observed in the last 100 m between the 800 and the 1500 m efforts are due to the difference in speeds and the fact that the 1500 m effort is run at a steady-state pace just above vVO_2max_, whereas the 800 m is an all-out effort [1].

The highest Tlim at VO_2max_%Ttot measured was in the 3000 m effort, while the lowest was measured in the marathon. Indeed, the 3000 m runners spent half of their time at VO_2max_ (51 ± 18% of Ttot), while the marathon runners reached VO_2max_, but only for 5% of the time.

Maron et al. confirmed that VO_2max_ was reached during 4% of the marathon in his research using Douglas bags [10]. We recently analyzed the pacing strategy of the world record marathon performance of Eluid Kipchoge at the 2019 Berlin marathon, 2h01 [43]. Kipchoge implemented a fast start near vVO_2max_, then allowed himself to “recover” during the following two-thirds of the marathon by running below his threshold and running above vVO_2max_ km before the finish [43]. Many marathons are now won in a final sprint; Kenya’s Lawrence Cherono won the 2019 Boston Marathon in such a manner.

The 3000 m effort is a true balance between aerobic and anaerobic contributions, with high energy production at VO_2max_. This corresponds to the average power at which the longest time to exhaustion at VO_2max_ is obtained, based on a model of the maximal endurance time at VO_2max_ [2] and experimental data from 90% to 140% of vVO_2max_ [12,44].

This relative endurance time spent at VO_2max_ was only a factor of performance in the group for which the Tlim at VO_2max_%Ttot was the highest and the lowest, i.e., the 3000 m and marathon, respectively). Indeed, the 3000 m effort was the distance eliciting the highest Tlim at VO_2max_%Ttot (more than half of the time), and the race for which the Tlim at VO_2max_%Ttot was significantly correlated with the performance.

Previously, our laboratory studied the concept of time spent at VO_2max_ by observing the speeds that elicit the longest time to exhaustion at VO_2max_ [44,45]. However, we now appreciate that this approach is flawed because it was based upon the model of constant power or speed, and not according to variable pace running. It would be better to study this concept using variable pace running, which is how humans run naturally. Indeed, the time spent at vVO_2max_ was accurately predicted when the vVO_2max_ was expressed as a percentage of the maximal speed reserve (i.e., the difference between maximal sprint velocity and the “critical speed” [44]. In our study, the average speed during the 3000 m was the closest to the critical speed at VO_2max_. This “critical speed” is that speed between at which vVO_2max_ and maximal lactate are reached. This is significant because critical speed corresponds to the highest metabolic rate at which energy is supplied through substrate-level phosphorylation and reaches a steady-state at VO_2max_. The critical speed represents the highest metabolic rate at which the energy supply produced via substrate-level phosphorylation reaches a steady-state below VO_2max_, and represents the greatest rate of energy production via “pure oxidative” just above the maximal lactate steady state [46,47].

However, this critical speed model was developed to find the speed that elicits the maximal time spent at VO_2max_. Billat et al. (1999) developed the concept of the critical speed at VO_2max_ (CP’) and defined it as the speed that can be maintained while running at VO_2max_ [45]. The authors used a test with progressively increasing speeds to determine the subjects’ vVO_2max_, which is defined as the speed at which VO_2max_ is attained.

Therefore CP’, i.e., the speed eliciting the maximal time spent at VO_2max_, was higher than the traditional critical speed and was then defined as the speed between the velocity at maximal lactate steady state and vVO_2max_ (equal to 87% of vVO_2max_ in Morton and Billat, 2000). Therefore, CP’ was sufficient to drive VO_2_ to its maximum and elicit the maximal time before exhaustion [2]. Expressing running intensity as a percentage of the difference between maximal velocity (measured from an individual 60 m effort) and the critical velocity allowed better prediction of the time limit at VO_2max_ compared to the critical speed VO_2max_ model [48]. This work confirmed prior studies performed on different exercises (swimming, cycling, kayaking, and running) by Faina et al. (1997), who have demonstrated that the anaerobic capacity was a significant factor of the time spent at VO_2max_ [49].

However, this approach was based on the constant speed paradigm. In addition, we know that interval training protocols, alternating speed above and below the critical speed, allow a doubling of the time limit at VO_2max_ in comparison with the time limit at vVO_2max_ (14 ± 5 vs. 4 ± 1 min) [50,51]. Surprisingly, extending this endurance time was shown to be possible using descending speed cardiorespiratory test protocols after having reached VO_2max_ until the maximal lactate steady state speed while maintaining VO_2max_ for almost 30 min [52].

## 5. Conclusions

In conclusion, our study showed that VO_2max_ is clearly elicited in all distances from the sprint to the marathon. A fast start and the time to reach VO_2max_ is important in increasing VO_2_ kinetics and to improve exercise tolerance. Human locomotion naturally uses a variable pace running strategy, and it is time to break down the barriers between the so-called aerobic and anaerobic metabolisms. We can only achieve this by moving the laboratory outdoors and performing studies in real-world environments and racing conditions. In this way, a new paradigm of applied physiology will be developed to provide new training and racing insights.

## Figures and Tables

**Table 1:** Descriptive physiological responses in UMTT.

Runners	*n*	vVO_2max_ (km∙h^−1^)	VO_2max_ (mL∙kg^−1^∙min^−1^)	HR_max_ (Beat·min^−1^)	RPE Last Stage of UMTT
100 m	13	15.4 ± 1.6	53.1 ± 5.5	196.3 ± 4.5	19.5 ± 0.5
200 m	13	15.4 ± 1.6	53.1 ± 5.5	196.3 ± 4.5	19.5 ± 0.5
800 m	8	19.3 ± 0.7 ^ab^	64.6 ± 3.4 ^ab^	196.9 ± 6.4	19.7 ± 0.5
1500 m	16	17.8 ± 2.2 ^ab^	59.0 ± 10.5	188.6 ± 12.6	19.8 ± 0.4
3000 m	9	16.2 ± 1.0 ^abc^	51.1 ± 5.3 ^cd^	181.9 ± 11.7 ^abc^	19.9 ± 0.3
10,000 m	7	19.1 ± 1.8 ^abe^	67.0 ± 6.5 ^abef^	183.4 ± 11.2 ^abc^	19.3 ± 0.5 ^de^
42,195 m	12	17.0± 0.9 ^abc^	55.4 ± 4.7 ^c^	189.1 ± 8.2 ^abc^	19.5 ± 0.5

Abbreviations: VO_2max_, maximal oxygen consumption; vVO_2max_, running speed associated with their maximal level of oxygen consumption maximal aerobic velocity; HR_max_, maximal heart rate and RPE, rating of perceived exertion and UMTT, University of Montreal Track Test. Note: ^a^ indicates a significant difference (*p* < 0.05) vs. 100 m, ^b^ 200 m, ^c^ 800 m, ^d^ 1500 m, ^e^ 3000 m and ^f^ marathon. The data are quoted as the mean ± SD.

**Table 2:** Performance (IAAF score and racing time), number of subjects having reached VO_2max_ and Tlim at VO_2max_ during the specific running distance.

Distance	*n*	IAAF Score	Race Time (hh:min:sec)	VO_2max_ Reached (*n*, %)	Tlim at VO_2max_ (s)	Tlim at VO_2max_%Ttot	Post-Run Lactate (mmol·L^−1^)
100 m	13	799.0 ± 143.5	11″ ± 0.5″	10 (76%)	3 ± 2.1	25.6 ± 18.5	14.0 ± 2.8
200 m	13	795.5 ± 135.5	23″ ± 1″1	11 (85%)	6 ± 4.0	28.5 ± 17.7	14.9 ± 1.5
800 m	8	563.0 ± 131.0 ^ab^	2′09″ ± 6″4 ^f^	8 (100%)	28 ± 19.7 ^aef^	22.0 ± 15.8	15.9 ± 1.7
1500 m	16	474.6 ± 191.8 ^ab^	4′40″ ± 24″7 ^acd^	15 (94%)	129 ± 92.2 ^abe^	41.7 ± 28.6	12.4 ± 1.8 ^bc^
3000 m	9	472.2 ± 218.8 ^ab^	10′07″ ± 1′9″ ^ab^	8 (89%)	341 ± 103.3 ^abcd^	51.4 ± 18.3 ^abc^	11.7 ± 2.3 ^bc^
10,000 m	7	522.4 ± 242.5 ^ab^	36′22″ ± 4′19″ ^ab^	7 (100%)	680 ± 590.6 ^abcd^	30.6 ± 27.2 ^f^	/
42,195 m	12	385.6 ± 190.7 ^ab^	3h7′17″ ± 18′41″ ^abcd^	10 (83%)	479 ± 497.9 ^abc^	4.1 ± 4.0 ^abcde^	6.6 ± 2.1 ^abcde^

Abbreviations: IAAF, International Association of Athletics Federations; VO2max, maximal oxygen consumption; Tlim, Time limit; Ttot, Total race time. Note: ^a^ indicates a significant difference (*p* < 0.05) vs. 100 m, ^b^ 200 m, ^c^ 800 m, ^d^ 1500 m, ^e^ 3000 m and ^f^ marathon. The data are quoted as the mean ± SD.
